# Effect of a bacteriophage *T5virus* on growth of Shiga toxigenic *Escherichia coli* and *Salmonella* strains in individual and mixed cultures

**DOI:** 10.1186/s12985-019-1269-7

**Published:** 2020-01-07

**Authors:** Yan D. Niu, Hui Liu, Roger P. Johnson, Tim A. McAllister, Kim Stanford

**Affiliations:** 1 Department of Ecosystem and Public Health, Faculty of Veterinary Medicine, University of Calgary, Calgary, AB T2N 1N4 Canada; 2 Lethbridge Research and Development Centre, Agriculture and Agri-Food Canada, Lethbridge, AB T1J 4B1 Canada; 3 National Microbiology Laboratory, Public Health Agency of Canada, Guelph, ON N1G 3W4 Canada; 4 Alberta Agriculture and Forestry, Agriculture Centre, Lethbridge, AB T1J 4V6 Canada

**Keywords:** Bacteriophages, *T5virus*, Biocontrol, Shiga toxigenic *Escherichia coli*, *Salmonella*

## Abstract

A previously isolated a bacteriophage, vB_EcoS_AKFV33 of *T5virus*, demonstrated great potential in biocontrol of Shiga toxigenic *Escherichia coli* (STEC) O157. This study further evaluated its potential as a biocontrol agent in broth culture against other important non-O157 serogroups of STEC and *Salmonella*. AKFV33 was capable of lysing isolates of STEC serogroups O26 (*n* = 1), O145 (n = 1) and *Salmonella enterica* serovars (*n* = 6). In a broth culture microplate system, efficacy of AKFV33 for killing STEC O26:H11, O145:NM and *Salmonella* was improved (*P* < 0.05) at a lower multiplicity of infection and sampling time (6–10 h), when STEC O157:H7 was also included in the culture. This phage was able to simultaneously reduce numbers of STEC and *Salmonella* in mixtures with enhanced activity (*P <* 0.05) against O157:H7 and O26:H11, offering great promise for control of multiple zoonotic pathogens at both pre and post-harvest.

## Background

Shiga toxin-producing *Escherichia coli* (STEC) and *Salmonella* are often carried by food-producing animals and remain leading causes of foodborne illness worldwide [1]. However, few effective on-farm interventions have been established. Moreover, with emergence of STEC and *Salmonella* that are resistant to conventional interventions (e.g. heat, acid and chemical sanitizers [1];), novel approaches are needed to control these pathogens in both primary and secondary food production. Bacteriophages (phages) are viruses that naturally use bacteria as hosts, and when virulent, induce lysis of the infected bacteria. Commercial phage-based products have been used in the biocontrol of important foodborne bacteria including STEC and *Salmonella* [2]. However, several challenges remain before phages could be widely used in the food industry. One major challenge is that the host range of phages is often limited to certain species and even strains within species. Although such specificity is often desirable, phage treatment to decontaminate foods adulterated with multiple pathogenic species would often require phage cocktails, a preparation including multiple phages with each targeting a specific pathogen. However, limitations in the formulation of phage cocktails such as interference among phages and high manufacturing costs [2] make the identification of polyvalent phages that kill multiple bacterial host species particularly desirable.

Previously, we identified and systematically characterized a phage vB_EcoS_AKFV33 (AKFV33), a *T5virus* that possesses many of the desired features of a biocontrol agent [3]. Moreover, we found AKFV33 to be superior to phages *T4virus, T1virus* and *rV5virus* used individually or as phage cocktails for inactivating O157 STEC on refrigerated beef [4]. Since several *T5virus* phages have shown a diverse host range including *Salmonella,* non-O157 serogroups of STEC and *Shigella* [5–10], we hypothesized that AKFV33 may have lytic activity against other serogroups of STEC and *Salmonella* strains. Consequently, the objective of this study was to evaluate the efficacy of AKFV33 in biocontrol of several selected serogroups of STEC and various *Salmonella* serovars in a broth culture system*.*

## Methods

### Phage microplate virulence assay

Host range and lytic activities of phage AKFV33 were assessed using a microplate phage virulence assay [11]. High titer phage stocks (> 10^9^ plaque forming units (PFU)/ml) were propagated and filter-purified as previously described [3]. To estimate multiplicity of infection (MOI), the filter-purified phage stocks were serially diluted and incubated at 37 °C without shaking for 5 h with 10-fold diluted overnight cultures of bacteria in a 96-well microplate. After incubation, wells were examined visually for turbidity and the highest dilution that resulted in complete lysis (no discernable turbidity) of bacteria was recorded. The MOI for each phage-host assay was calculated by dividing the initial number of phages in the greatest-dilution wells by the initial number of bacteria added, as determined from plate counts of serially diluted bacterial cultures. Sensitivity to phages was categorized as follows: extremely susceptible: (10^− 6^ ≤ MOI < 10^− 2^); highly susceptible: (0.01 ≤ MOI < 1); moderately susceptible: (1 ≤ MOI < 10); and minimally susceptible: (10 ≤ MOI < 100).

### Phage lysis kinetics

To further assess dynamics of AKFV33 infection, a bacterial growth inhibition curve was conducted. Phage stocks (~ 10^8^ PFU∙ml^− 1^, 20 μl) were serially diluted in 96-well microplates and incubated individually for 10 h at 37 °C with diluted overnight bacterial cultures (~ 10^4^ colony forming units (CFU)∙ml^− 1^, 20 μl; Table 1), at final MOIs of 0.01, 0.1, 1, 10, 100 and 1000, respectively. Mixtures of the selected STEC and *Salmonella* strains (Table 1, ~ 10^4^ CFU ml^− 1^ in total) were also set up in the same microplates and inoculated with AKFV33 at the same MOIs. Untreated control wells with only the bacteria in mTSBY (tryptic soy broth with 10 mmol l^− 1^ MgSO_4_ and 0.6% yeast extract), and blank control wells containing only mTSBY were included in each microplate. The plates were incubated at 37 °C and the optical density (OD_600nm_) was read at 2 h intervals over 10 h using a SynergyTM HT multi-mode microplate reader (BioTek, Winooski, VT, USA). Two independent experiments were performed in duplicate. The blank values were subtracted from absorbance measures at 600 nm, to give a final corrected optical density.

**Table 1:** Effect of phage AKFV33 treated individual and mixture of STEC and *Salmonella* at different MOIs

Species/Serotypes	Bacterial	Source	Susceptibility^1^	Bacterial individual or mixed culture	Mean OD_600nm_ at each MOI^2^							
	strains #		(MOI value)		Phage-free Control	0.01	0.1	1	10	100	1000	Across MOIs
STEC O157:H7	R508N	Bovine	9 × 10^−6^	Individual	0.261	0.006a	0.004a	0.001a	0.001a	0.001a	0a	0.002
STEC O26:H11	EC19960464	Bovine	6 × 10^−5^	Individual	0.310	0.05a	0.011b	0.004b	0.002b	0b	0b	0.011
STEC O145:NM	EC19970355	Human	4 × 10^−3^	Individual	0.224	0.156a	0.067b	0.067b	0.02c	0.015c	0.003c	0.055
*S.* I 4, [5],12:i	20,104,603	Porcine	4	Individual	0.162	0.165	0.094a	0.097a	0.063a	0.063a	0.048b	0.088
*S.* Typhimurium	ATCC14028	Porcine	3	Individual	0.262	0.227	0.207	0.220	0.174	0.221	0.038a	0.181
				O26:H11 + O157:H7	0.315	0.013a^*^	0.005a	0.006a	0a	0.001a	0a	0.004
				O145:NM + O157:H7	0.268	0.009a^(***)^	0.005a^(**)^	0.004a^(**)^	0.003a	0.005a	0.003a	0.005
				O26:H11 + O145:NM + O157:H7	0.316	0.014a^*(***)^	0.004b^(**)^	0.005b^(**)^	0.001b	0.002b	0.002b	0.005
				*S*. I 4, [5],12:i + O157:H7	0.230	0.099a^**^	0.002c^***^	0.064b^**^	0.004c^**^	0.012c^**^	0.003c^*^	0.031
				*S*. Typhimurium + O157:H7	0.262	0.037a^(***)^	0.015a^(***)^	0.014a^(***)^	0.008a^(***)^	0.009a^(***)^	0.007a	0.015
				*S.* I 4, [5],12:I + *S.* Typhimurium + O157:H7	0.228	0.004a^***(***)^	0.004a^***(***)^	0.011a^***(***)^	0.008a^**(***)^	0.008a^**(***)^	0.008a^*^	0.007

^1^Susceptibility of strains to phages were determined by microplate phage virulence assay for each MOI (Multiplicity of infection) value

^2^The blank values were subtracted from absorbance measures at 600 nm, to give a final corrected optical density. Mean OD_600nm_ at each MOI were calculated by averaging OD_600nm_ from 2 h, 4 h, 6 h, 8 h and 10 h

Letters which differ after the mean values indicate differences (*P* < 0.05) among MOIs within each bacterial culture

Asterisks^*^, ^**^ and ^***^ indicate a statistical difference between phage-treated individual and mixed culture within same MOI at *P* < 0.05, *P <* 0.01 and *P* < 0.001, respectively

### Enumeration of bacteria

To determine if efficacies of AKFV33 against non-O157 STEC and *Salmonella* were repeatable in larger-scale broth cultures, individual and 3 mixed overnight cultures of STEC O157:H7 R508N, O26:H11 EC19960464 and *S.* Typhimurium ATCC14028 (1 mL, ~ 10^5^ CFU ml^− 1^) were inoculated with AKFV33 at ~ 10^9^ PFU ml^− 1^ (MOI = 10^4^) in 9 mL of mTSBY and incubated at 37 °C with shaking at 170 rpm. Subsamples (1.8 mL) for enumeration of the inoculated strains were withdrawn at 4, 7, 10 and 24 h and centrifuged. Pellets were re-suspended in sterile PBS (phosphate-buffered saline), serially diluted and plated on tryptic soy agar with 50 g/ml nalidixic acid (Sigma, Oakville, ON, Canada; O157:H7), Rhamnose MacConkey (Innovation Diagnostics, Saint-Eustache, QC, Canada; O26:H11) and brilliant green agar (Oxoid, Toronto, ON, Canada; *S.* Typhimurium). Two independent experiments were performed in duplicate.

### Statistical analysis

Results from phage lysis kinetics and enumeration of bacteria from larger scale broth cultures were compiled from two independent experiments, respectively. The OD values at 600 nm were square-root transformed and colony forming units were log-transformed. Influence of MOIs and time on phage efficacy were analyzed using the MIXED model with repeated measure. Least-squares were used to differentiate means (*P* < 0.05). The analyses were conducted with SAS (version 9.4, SAS Institute, Cary, NC).

## Results

Of 36 non-O157 STEC strains including clinically important serogroups O26, O45, O91, O103, O111, O113, O121, O128 and O145 (*n* = 4 per serogroups), only STEC O26:H11 strain EC19960464 and O145:NM strain EC19970355 were extremely susceptible to AKFV33 at MOIs of 6 × 10^− 5^ and 4 × 10^− 3^, respectively. Of 39 *Salmonella* strains representing *Salmonella enterica subspp. Enterica* serovars Typhimurium, Enteritidis, Heidelberg, I 4 [5],12:i-, Saintpaul, Newport, Infantis, Hadar, Ago, Kumasi, Landau, Soerenga and Urbana (*n* = 1–5 per serovar, Additional file 1: Table S1), only 1 of 5 *S.* Typhimurium strains (ATCC14028), 2 of 5 *S.* I 4 [5],12:i- strains (20104603 and 20085085), 1 of 1 *S.* Kumasi strain (20015671) and 1 of 1 *S.* Landau strain (20015670) were highly or moderately susceptible to AKFV33 with MOIs ranging from 0.5 to 4. Although complete lysis of other strains was not observed after 5 h of phage treatment, phage-treated cultures (*n* = 3, 1 and 1, respectively), from STEC O26, *S.* Ago and *S.* Soerenga showed complete lysis at MOI = 2–10 at 2 h (data not shown). The subsequent re-growth after 2 h may indicate rapid emergence of phage-resistant mutant strains in these cultures, which complies with previous studies of other *T5virus* strains [6, 7]. The ability of AKFV33 to lyse some non-O157 STEC and *Salmonella* strains is consistent with other reports that *T5viruses* may have broad host ranges across multiple bacterial species [5–10]. The susceptibility of the *Salmonella* serovars Ago, Kumasi, Landau and Soerenga strains in the present study may be explained at least in part by their somatic (O) antigens, which are important phage receptors in Gram-negative bacteria [12]. All these serovars possess the Group N O30 *Salmonella* antigen, which is antigenically strongly related to the O157 antigen of *E. coli* [13], and hence may enable binding of the O157-infecting phage AKFV33 to these *Salmonella* serovars.

**Fig. 1:**
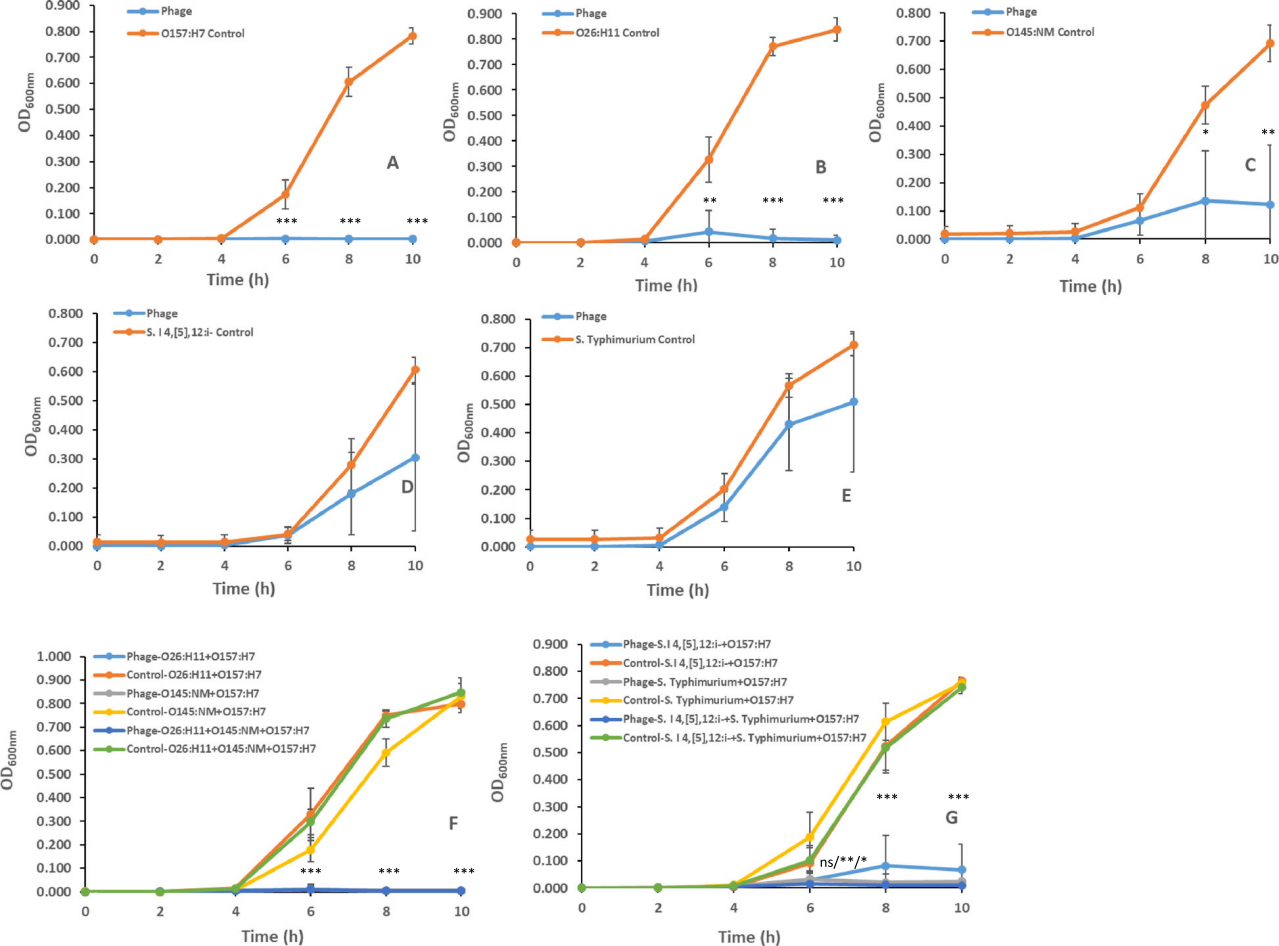
Growth curves of selected STEC and *Salmonella* strains in individual and mixed cultures treated and not treated with phage AKFV33 across MOIs. **a** O157:H7 R508N; **b** O26:H11 EC19960464; **c** O145:NM EC19970355; **d** *S.* I 4, [5],12:I- 20104603; **e** *S.* Typhimurium ATCC14028; **f** Mixture of STEC O157:H7 R508N, O26:H11 EC19960464 and O145:NM EC19970355; **g** Mixture of STEC O157:H7 R508N and *Salmonella S.* I 4, [5],12:I- 20104603 and *S.* Typhimurium ATCC14028*.* Bars present standard deviations. Asterisks^*^, ^**^ and ^***^ indicate a statistical difference of OD_600nm_ value within same sampling time between phage-treated and non-treated individual or mixed culture at *P* < 0.05, *P* < 0.01 and *P* < 0.001, respectively. For (**f**) and (**g**), ^***^ indicates significance evident in all phage-treated 3 pathogen mixtures; For (**g**), at 6 h, ns indicates OD_600nm_ value did not differ between phage-treated and non-treated 2 mixture of *S.* I 4, [5],12:I- + O157:H7, whereas ^*^ and ^**^, respectively, indicate OD_600nm_ value differed between phage-treated and non-treated mixtures of *S.* Typhimurium + O157:H7 and mixtures of 3 pathogenic bacteria

Across MOIs and times, AKFV33 inhibited growth of all the individual bacteria and their mixtures with OD_600nm_ ranging from 0.002 to 0.181(*P* < 0.05; Table 1). Moreover, AKFV33 at MOI < 10 was more efficient at lysing STEC O145:NM strain EC19970355 (*P* < 0.01) in mixtures (O145:NM + O157:H7 and O26:H11 + O145:NM + O157:H7) than in individual cultures, and at MOI =0.01, phage activity against STEC O26:H11 strain EC19960464 in mixed cultures (O26:H11 + O157:H7 and O26:H11 + O145:NM + O157:H7 was also increased (*P* < 0.05). AKFV33 exhibited less activity against *Salmonella* than against STEC, inhibiting growth of *S.* I 4, [5],12:i- strain 20104603 at MOI > 0.01 (*P* < 0.001) and *S.* Typhimurium ATCC14028 at MOI = 1000 (*P* < 0.05). However, in 2 mixtures (*S.* I 4, [5],12:i- + O157:H7 and *S.* Typhimurium + O157:H7), activity of AKFV33 against *Salmonella* was substantially greater, as growth of *S.* I 4, [5],12:I- strain 20104603 (*P* < 0.05) was reduced at all MOIs and *S.* Typhimurium strain ATCC14028 (*P* < 0.001) at MOI < 1000. Furthermore, this enhanced phage activity was also evident in a 3 bacterial mixed culture (*S.* I 4, [5],12:i- + *S.* Typhimurium + O157:H7). In addition, AKFV33 was more active against O26:H11 (6 h) and O145:NM (6, 8 and 10 h), *S.* I 4, [5],12:i- (8 and 10 h for mixture of two pathogens, 6, 8 and 10 h for 3 a mixture of two pathogens) and *S.* Typhimurium (6, 8 and 10 h) across all MOIs (*P* < 0.001) in mixed cultures containing STEC O157:H7 than in individual cultures of these strains without STEC O157:H7 (Fig. 1).

**Fig. 2:**
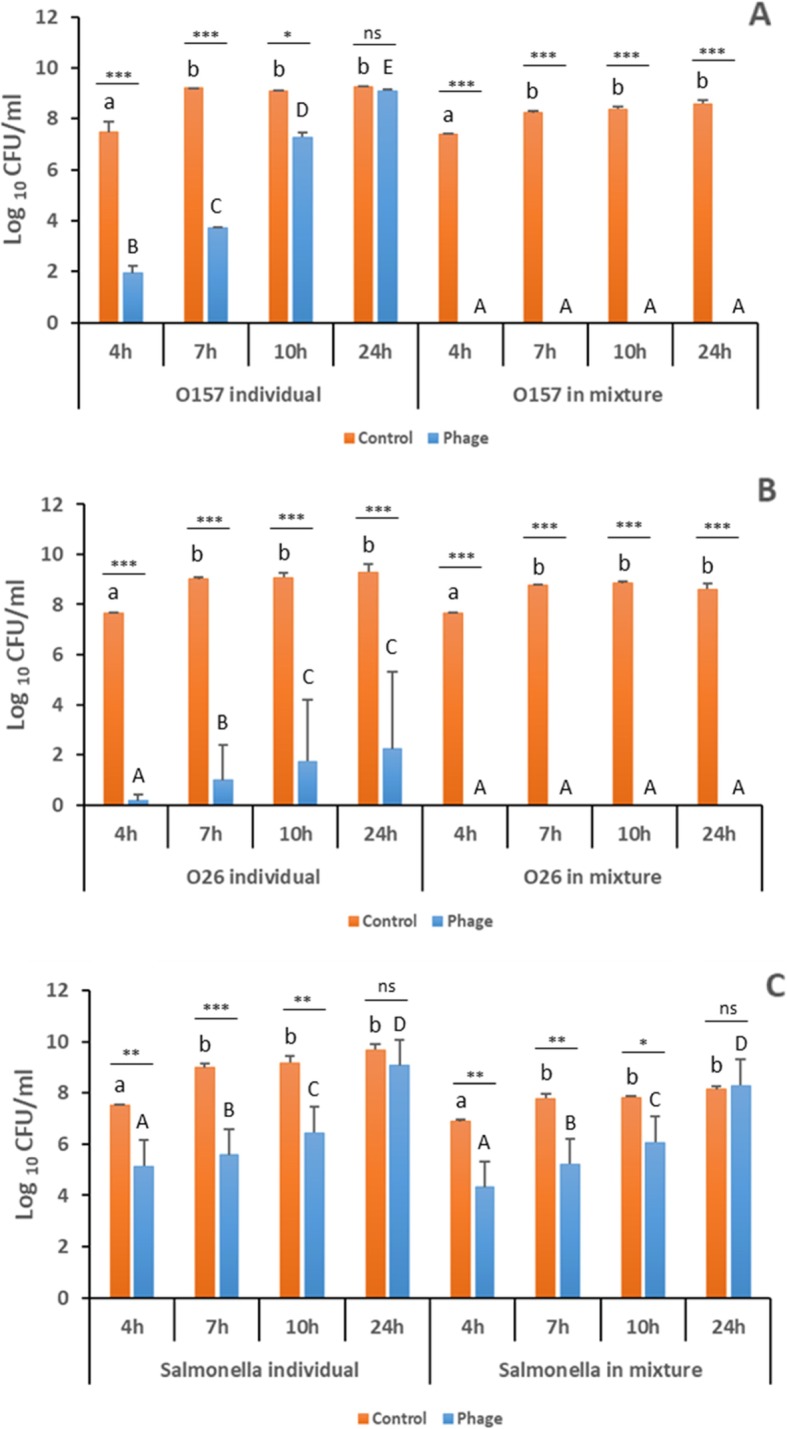
Effect of phage AKFV33 at a MOI of 10^4^ on numbers of selected STEC and *Salmonella* strains grown in larger-scale individual and 3 bacterial mixed cultures. **a** STECO157:H7 R508N; **b** STECO26:H11 EC19960464; **c** *S.* Typhimurium ATCC14028. Bars present standard deviation. Asterisks^*^, ^**^ and ^***^ indicate a statistical difference of bacterial numbers between phage-treated and non-treated individual or mixed culture at *P* < 0.05, *P* < 0.01 and *P* < 0.001, respectively, whereas “ns” means no statistical difference (*P* > 0.1). Lowercase and uppercase letters represent that bacterial numbers differ (*P <* 0.05) overtime between individual and mixed culture treated with (**A**-**E**) or without phages (**a**, **b**)

For individual bacterial cultures, AKFV33 caused an overall reduction of 7.5 ± 0.4 log_10_ CFU/ml in O26:H11, greater (*P* < 0.001) than those in STEC O157:H7 (2.5 ± 2.7 log_10_ CFU/ml) or *S.* Typhimurium (2.2 ± 1.2 log_10_ CFU/ml, Fig. 2). The greatest efficacy of the phage (*P <* 0.001) was at 4 and/or 7 h, but was reduced (*P* < 0.001) thereafter. Notably, after 24 h of incubation, phage treatment had no effect (*P >* 0.1*)* on the numbers of O157:H7 or *S.* Typhimurium*.* When exposed to a mixture of O157:H7, O26:H11 and *S.* Typhimurium, AKFV33 was able to simultaneously reduce (*P* < 0.01) numbers of each bacteria in the mixtures by 2–8 log_10_ CFU/ml (Fig. 2). Moreover, both O157:H7 and O26:H11 in the mixture were undetectable (< 300 CFU/ml) at each sampling time, even after 24 h. This indicates that AKFV33 was more active and/or the targeted STEC were more vulnerable to the phages (*P* < 0.05) in mixed cultures. In contrast, *S.* Typhimurium was equally sensitive to the phages either alone or in a mixture with O157:H7.

## Discussion

To our knowledge, this is the first study to evaluate effectiveness of a polyvalent phages *T5virus* in control of STEC and *Salmonella* in a mixed culture. In our previous studies, AKVF33 was shown to be highly virulent to various phage types of STEC O157 strains [3], but its virulence for other foodborne pathogens was unknown. Here we have found that AKVF33 is virulent for a broad host range that includes some non-O157 STEC and *Salmonella* serovars, and that in mixed cultures, AKVF33 not only simultaneously reduces numbers of STEC and *Salmonella*, but in some instances also has greater efficacy. Further study is required to understand mechanism(s) underlying this improved efficacy. Potentially, replication of AKVF33 in a preferred host (O157:H7) and enhanced concentrations of phage led to improved control of non-preferred hosts (*Salmonella* and non-O157 *E. coli*). In addition, this finding was consistent with earlier reports that phage av-08 (unknown taxonomy) was able to decontaminate *S.* Montevideo and STEC O157:H7 on chicken skin [14]. Costa et al. [15] also found that single phage ELY-1 or phSE-5 (unknown taxonomy) reduced number of non-O157 *E. coli* and *S. Typhimurium* ATCC13311 in a mixture, although this reduction was less than produced by a cocktail of both of these phages in broth culture. The relative contribution of polyvalent phages vs phage cocktails to bacterial biocontrol remains unclear. However, Zhao et al. [16] reported that a polyvalent phage of the *Siphoviridae* was effective in decreasing population of *E. coli* K12 and *Pseudomonas aeruginosa* in a soil-carrot system. Although less effective than a cocktail of phages against these organisms, polyvalent phages were more capable than the phage cocktail of sustaining the diversity of the commensal bacterial community in the system. In another study, a polyvalent phage of the *Podoviridae* in combination with biochar treatment effectively eliminated *E. coli* K12 and *P. aeruginosa* in a soil-lettuce system, while synergistically enhancing indigenous bacterial communities [17]. This suggests that polyvalent phages such as AKFV33 may be used for simultaneous inhibition of various zoonotic bacterial pathogens without harming beneficial microbes resident in gastro-intestinal tracts of food animals or in food products.

## Supplementary information

**Additional file 1 : Table S1.** Host range and lytic activity of Phage AKFV33 against *Salmonella* strains

## Data Availability

Not applicable.

## Abbreviations

MOI: Multiplicity of infection
mTSB: Tryptic soy broth with 10 mmol l^-1^ MgSO4 and 0.6% yeast extract
OD: Optical density
PBS: Phosphate-buffered saline
STEC: Shiga toxigenic *Escherichia coli*
