# Supplementary material for: Effect of a bacteriophage T5virus on growth of Shiga toxigenic Escherichia coli and Salmonella strains in individual and mixed cultures
Source: Virol J. 2020 Jan 7;17:3. doi: 10.1186/s12985-019-1269-7 (PMC6947960; doi:10.1186/s12985-019-1269-7)
Supplement: Supplementary file 1 — Additional file 1 : Table S1. Host range and lytic activity of Phage AKFV33 against Salmonella strains [file 12985_2019_1269_MOESM1_ESM.docx]

Additional file 1: table S1. Host range and lytic activity of Phage AKFV33 against *Salmonella* strains

| **Strain #** | **Serovar/Antigen** | **Animal Origin** | **Isolation** | **Phage susceptibility** |
| --- | --- | --- | --- | --- |
|  |  |  | **Source** | **(MOI value)** |
| 20001498 | Typhimurium/4,5:i:1,2 | Bovine | ON |  |
| 19981565 | Typhimurium/4,5:i:1,2 | Bovine | QC |  |
| 20091322 | Typhimurium/4,5:i:1,2 | Bovine | ON |  |
| 20105435 | Typhimurium/4,5:i:1,2 | Bovine | ON |  |
| ATCC 14028 | Typhimurium/4,5:i:1,2 | Chicken | N/A | 3 |
| 20103417 | Enteritidis/9,12:g,m:- | Chicken | AB |  |
| 20100112 | Enteritidis/9,12:g,m:- | Chicken | NS |  |
| 20084000 | Enteritidis/9,12:g,m:- | Chicken | ON |  |
| 19970903 | Enteritidis/9,12:g,m:- | Chicken | QC |  |
| 20101731 | Heidelberg/4:r:1,2 | Chicken | SK |  |
| 20100150 | Heidelberg/4:r:1,2 | Chicken | NS |  |
| 20104948 | Heidelberg/4:r:1,2 | Chicken | BC |  |
| 20101068 | Heidelberg/4:r:1,2 | Chicken | AB |  |
| 20104643 | Heidelberg/4:r:1,2 | Chicken | PEI |  |
| 20101268 | I 4 [5],12:i:-/4,5:i:- | Porcine | QC |  |
| 20104603 | I 4 [5],12:i:-/4,5:i:- | Porcine | SK | 4 |
| 20085085 | I 4 [5],12:i:-/4,5:i:- | Porcine | MB | 4 |
| 20100108 | I 4 [5],12:i:-/4,5:i:- | Bovine | ON |  |
| 20111874 | I 4 [5],12:i:-/4,5:i:- | Bovine | ON |  |
| 19971313 | Saintpaul/4:e,h:1,2 | Turkey | BC |  |
| 20053559 | Saintpaul/4:e,h:1,2 | Turkey | QC |  |
| 20070554 | Newport/6,8:e,h:1,2 | Porcine | SK |  |
| 20114211 | Newport/6,8:e,h:1,2 | Turkey | ON |  |
| 20103229 | Newport/6,8:e,h:1,2 | Water | AB |  |
| 20011809 | Newport/6,8:e,h:1,2 | Bovine | NB |  |
| 20104604 | Infantis/6,7:r:1,5 | Porcine | MB |  |
| 20102119 | Infantis/6,7:r:1,5 | Porcine | SK |  |
| 20104946 | Infantis/6,7:r:1,5 | Chicken | BC |  |
| 20100138 | Infantis/6,7:r:1,5 | Chicken | NS |  |
| 20094741 | Infantis/6,7:r:1,5 | Bovine | ON |  |
| 19982620 | Hadar/6,8:z10:x | Chicken | QC |  |
| 19993558 | Hadar/6,8:z10:x | Chicken | ON |  |
| 19993405 | Hadar/6,8:z10:x | Chicken | ON |  |
| 19971368 | Hadar/6,8:z10:x | Turkey | BC |  |
| 20094925 | Ago/30:z38:- | Unknown | ON |  |
| 20015671 | Kumasi/30:z10:z15 | Unknown | ON | 0.5 |
| 20015670 | Landau/30:i:2 | Unknown | ON | 1 |
| 20075706 | Soerenga/30:i:l,w | Unknown | QC |  |
| 20004763 | Urbana/30:b:x | Porcine | QC |  |

^1^ ON: Ontario; QC: Quebec; AB: Alberta; NS: Nova Scotia; SK: Saskatchewan; BC: British Columbia; PEI: Prince Edward Island; MB: Manitoba; NB: New Brunswick; N/a: Not applicable.

^2^ Susceptibility of strains to phages were determined by microplate phage virulence assay

and presented by MOI (Multiplicity of infection) value.
